# Short-Term Synaptic Plasticity in the Dentate Gyrus of Monkeys

**DOI:** 10.1371/journal.pone.0020006

**Published:** 2011-05-20

**Authors:** Ryoi Tamura, Hiroshi Nishida, Satoshi Eifuku, Kaoru Nagao, Hiroaki Fushiki, Yukio Watanabe, Taketoshi Ono

**Affiliations:** 1 Department of Integrative Neuroscience, Graduate School of Medicine and Pharmaceutical Sciences for Research, University of Toyama, Toyama, Japan; 2 Otorhinolaryngology, Graduate School of Medicine and Pharmaceutical Sciences for Research, University of Toyama, Toyama, Japan; 3 Judo Neurophysiotherapy, Graduate School of Medicine and Pharmaceutical Sciences for Research, University of Toyama, Toyama, Japan; Tokyo Medical and Dental University, Japan

## Abstract

The hippocampus plays an important role in learning and memory. Synaptic plasticity in the hippocampus, short-term and long-term, is postulated to be a neural substrate of memory trace. Paired-pulse stimulation is a standard technique for evaluating a form of short-term synaptic plasticity in rodents. However, evidence is lacking for paired-pulse responses in the primate hippocampus. In the present study, we recorded paired-pulse responses in the dentate gyrus of monkeys while stimulating to the medial part of the perforant path at several inter-pulse intervals (IPIs) using low and high stimulus intensities. When the stimulus intensity was low, the first pulse produced early strong depression (at IPIs of 10–30 ms) and late slight depression (at IPIs of 100–1000 ms) of field excitatory postsynaptic potentials (fEPSPs) generated by the second pulse, interposing no depression IPIs (50–70 ms). When the stimulus intensity was high, fEPSPs generated by the second pulse were depressed by the first pulse at all IPIs except for the longest one (2000 ms). Population spikes (PSs) generated by the second pulse were completely blocked or strongly depressed at shorter IPIs (10–100 or 200 ms, respectively), while no depression or slight facilitation occurred at longer IPIs (500–2000 ms). Administration of diazepam slightly increased fEPSPs, while it decreased PSs produced by the first pulse. It also enhanced the facilitation of PSs produced by the second stimulation at longer IPIs. The present results, in comparison with previous studies using rodents, indicate that paired-pulse responses of fEPSPs in the monkey are basically similar to those of rodents, although paired-pulse responses of PSs in the monkey are more delayed than those in rodents and have a different sensitivity to diazepam.

## Introduction

The hippocampus is important for certain classes of learning and memory [1]–[3]. On a mechanistic level, synaptic plasticity in the hippocampus is hypothesized to be a neural substrate of the mnemonic process [4], [5]. There are several forms of synaptic plasticity, including short-term and long-term types. Paired-pulse stimulation is a standard technique for the evaluation of short-term synaptic plasticity, and its effects in the hippocampus and their underlying mechanisms have been intensively investigated using rodents.

It is known that paired-pulse facilitation (PPF) and paired-pulse depression (PPD) appear in the dentate gyrus of rats in a manner dependent on stimulation sites, stimulus intensities and inter-pulse intervals (IPIs) [6]–[11]. At low stimulus intensities that do not produce firing of neurons, paired-pulse responses are thought to reflect mainly presynaptic functions, while at high stimulus intensities producing neuronal firing, additional effects such as recurrent inhibition, feed forward inhibition and recurrent excitation participate in the response [12]–[16]. The paired-pulse test has often been used in the dentate gyrus of rats to characterize the effect of anesthetic or antianxiety drugs on GABAergic inhibition e.g. [12], [13], [17].

Although paired-pulse responses in the hippocampus have been well characterized in rodents, very limited data of this kind are available for the primate hippocampus. We have recently developed an animal model for *in vivo* recording of evoked local field potentials (LFPs) in the monkey hippocampus [18]. Therefore, in the present study, we tested the effect of paired-pulse stimulation on LFPs recorded in the dentate gyrus of monkeys. Furthermore, we investigated the influence of an antianxiety drug, diazepam, on the paired-pulse effects to study the role of GABAergic inhibition on this type of short-term synaptic plasticity.

## Materials and Methods

### Subjects

In the present study, we use two male monkeys (*Macaca fuscata*; 11.0 and 5.0 kg at the beginning of the experiment). The animals were treated in strict compliance with the Animal Care and Use Committee of University of Toyama, and with the NIH Guide for the Care and Use of Laboratory Animals. The protocol was approved by the Animal Care and Use Committee of University of Toyama (Permit Number: S-2009 MED-2). All studies were performed in accordance with the recommendations of the Weatherall report, “The use of non-human primates in research”. All surgery was performed under sodium pentobarbital anesthesia, and all efforts were made to minimize suffering. To reduce pain, a nonsteroidal anti-inflammatory drug was administered once or twice a day after surgery for a few days (flunixin meglumine, 1 mg/kg, i.m.). Each monkey was allowed to recover from invasive manipulations for an appropriate period of time as described below.

### Cranioplastic surgery and electrode implantation

The surgical procedure was the same as that described in our previous study [18]. Briefly, a cranioplastic cap (a head-restraining device) was surgically fixed to the skull using titanium screws under sodium pentobarbital anesthesia (35 mg/kg, i.m.). To prevent infection, the surgical operations were performed under an aseptic condition, and an antibiotic was administered systemically before and after surgery (orbifloxacin, 5 mg/kg, i.m.). After a recovery period of more than 2 weeks following the surgery, the head of the monkey was again fixed to the stereotaxic device through the cap under sodium pentobarbital anesthesia, and a marker rod (tungsten, 0.5 mm in diameter) was stereotaxically inserted in the brain with its tip placed at a known coordinate (near to the dentate gyrus on the brain atlas [19]). The rod was fixed to the cranioplastic cap using dental acrylic. The head of the monkey was released from the stereotaxic device and magnetic resonance (MR) images and X-ray photographs were taken. Based on these MR images and X-ray photographs, the insertion coordinates of stimulation and recording electrodes were determined. In the present study the stimulation and recording were performed using concentric electrodes consisting of an enamel-coated stainless steel wire encased in a polyurethane-coated stainless steel cannula. The stimulation and recording electrodes were advanced into the hemisphere using two micromanipulators, aiming at positions close to the expected coordinates of the medial part of the perforant path (17 mm anterior to the interaural line, 12 mm lateral to the midline) and the dentate gyrus (12 mm anterior to the interaural line, 14 mm lateral to the midline), respectively. The stimulation electrode was connected to a stimulator (SEN-7203, Nihon Kohden) through an isolation unit (SS202J, Nihon Kohden); the recording electrode was connected to a main amplifier (Lynx-8, Neuralynx) through a high input impedance head amplifier made of dual FET (K389, Toshiba). A microcomputer equipped with a multifunction board (DT3010, Data Translation) gave rise to a trigger signal to the stimulator every 10 s; the stimulator output a single positive square pulse of 0.2-ms duration synchronized to the trigger signal. We usually set the stimulus intensity to a level between 100 and 200 µA during the optimization of electrode positions but increased it if necessary. The output of the main amplifier was monitored on a storage oscilloscope. Under this electrophysiological monitoring, electrodes were further advanced ventrally and evoked LFPs were recorded to produce depth profiles. This procedure was repeated, shifting the insertion coordinates of the recording and/or stimulation electrodes (for details, see our previous study [18]). Based on these depth profiles, both electrodes were placed at the most suitable coordinates of the target brain structures (i.e., the medial part of the perforant path for stimulation, and the hilar region of the dentate gyrus for recording). These electrodes were then fixed to the cranioplastic cap by dental acrylic.

### Stimulus intensity-response relationships

After one week of recovery from the electrode implantation, the monkey, sitting in a primate chair, was transferred to a recording room. The stimulator and amplifier lines were connected to the electrode lines on the cranioplastic cap in the same configuration as used in the electrode implantation. The monkey was then placed in a cage to record evoked LFPs in a freely-behaving condition. To acquire stimulation intensity-response relations, evoked LFPs were recorded while gradually increasing the stimulus intensity in a range from 10 to 1000 µA (the pulse parameters were the same as those used in the electrode implantation). Stimulation was repeated 5 times at each stimulus intensity at 0.1 Hz. From these data, stimulus intensity-response curves were generated for field excitatory postsynaptic potential (fEPSP) slopes and population spike (PS) amplitudes.

### Paired-pulse test

Following the recording of the stimulus intensity-response relationships, we performed the paired-pulse test under a condition with or without administration of diazepam. In this test, we applied a pair of pulses to the medial part of the perforant path with varying IPIs between 10–2000 ms (10, 20, 30, 50, 70, 100, 200, 500, 1000 or 2000 ms), where the stimulation parameters of each pulse were the same as those used in the electrode implantation. The pair of pulses was given repeatedly every 10 s (0.1 Hz). We chose these IPIs because similar IPI sequence was used in many previous studies on paired-pulse responses in the dentate gyrus of rodents *in vivo* [e.g.], [ 7], [9]–[13], [17]. The low and high current intensities used in this test were determined according to the method reported by Joy and Albertson [9]: for low intensity stimulation, it was set at an intensity halfway between fEPSP and PS thresholds; for high intensity stimulation, it was set at an intensity evoking 80% of the maximum PS. To test the effect of diazepam (Cercine® Injection, Takeda), a drug used to potentiate the action of GABA on neurons in the central nervous system [20], on paired-pulse responses, a low or high dose (1.5 or 3.0 mg/kg, respectively) of this drug was injected into the monkey intramuscularly. These doses were determined based on previous studies in which the effect of diazepam on paired-pulse responses was examined in the hippocampus of rats *in vivo* [12], [13]. The paired-pulse test was initiated 10 min after the administration of diazepam, and completed within 35 min. One week elapsed between the low-dose and high-dose treatments.

In the paired-pulse test (regardless of the administration of diazepam), stimulus sequences, i.e., the order of IPIs and current intensities, were counter-balanced to cancel the effect of changes in behavioral states (e.g., levels of arousal) or diazepam levels in the blood. The stimulus sequence was repeated 10 times so that a total of 200 paired-pulse responses were recorded (10 IPIs×2 intensities×10 times) under each condition.

### Data storage and analysis

The signal from the main amplifier, monitored on the oscilloscope, was fed to the microcomputer through the multifunction board with a time resolution of 40 kHz/channel, shown on-line on a display and stored on a hard disk. Two parameters for LFP size (fEPSP slope and PS amplitude) were extracted from the waveform of individual LFPs or averaged data on five or ten successive evoked LFPs. The fEPSP slope (mV/ms) was measured as the inclination between 20% and 80% of the first positive peak amplitude. The PS amplitude (mV) was the distance (voltage) of a vertical line from the negative peak to a tangent line drawn between the PS onset and offset. The PS latency (ms) was the duration between the onset of stimulation and PS negative peak.

In the paired-pulse test, we calculated the ratios of the fEPSP slope, PS amplitude and PS latency of the evoked LFP for the first pulse to that for the second pulse (paired-pulse ratios). Facilitation and depression (PPF and PPD) were respectively quantified by paired-pulse ratio of fEPSP slope or PS amplitude. Subtraction correction was used whenever response components overlapped, such that the measurement points for the second response were occurring on a varying baseline due to the first response, which was especially the case at shorter IPIs. This was done by selecting a matching first response from the record with the longest IPI and subtracting that response from the paired-pulse record. This left a nearly “pure” second response whose measurement values (i.e., fEPSP slopes, PS amplitudes and PS latencies) could be accurately determined.

A paired *t*-test was performed on each data set of paired-pulse responses (i.e., the responses to the first pulse vs. those to the second pulse) using the measurement values of individual evoked LFPs. An appropriate analysis of variance (ANOVA) test was performed on data sets of the control and diazepam-treatment conditions, and statistically significant effects were further evaluated with Dunnet multiple comparisons (Systat, ver. 10). Statistically significant levels were set at *p*<0.05.

## Results

### Stimulus intensity-response relationships

**Figure 1:**
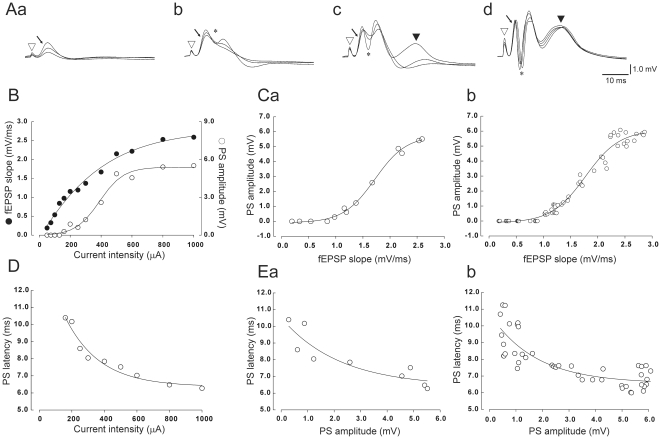
Stimulus intensity-response relationships. A: evoked local field potentials (LFPs) at different stimulus intensities (a: 50, 75 and 100 µA; b: 130, 160 and 200 µA; c: 250, 300 and 400 µA; d: 500, 600, 800 and 1000 µA). Each waveform is an average of 5 LFPs at each stimulus intensity. Inverted open triangles, stimulus artifacts; arrows, field excitatory postsynaptic potentials (fEPSPs); asterisks, population spikes (PSs); inverted filled triangles, positive deflection with a long latency following to clear PS. B: relationships between stimulus intensities and fEPSP slopes (filled circles) or PS amplitudes (open circles). C: relationships between fEPSP slopes and PS amplitudes of averaged (a) and individual (b) data. D: relationships between stimulus intensities and PS latencies. E: a relationship between PS amplitudes and PS latencies of averaged (a) and individual (b) data. Lines in B and C, sigmoid curves produced by Bolzmann fitting; lines in D and E, curves produced by exponential decay fitting.

When the stimulus intensity was gradually increased, evoked LFPs started to appear on the oscilloscope as a small positive deflection (fEPSP) at a stimulus intensity between 50–100 µA (Fig. 1 Aa and B). The evoked LFPs increased as the stimulus intensity increased: the slope of fEPSP became steeper, and a negative notch (PS) started to appear (Fig. 1 Ab and B). When the stimulus intensity was further increased, the fEPSP slope and PS amplitude also increased, and then each reached an asymptotic level (Fig. 1 Ac, d and B). In the stimulus intensity range that elicited PSs, a sigmoid relationship was observed between the fEPSP slopes and PS amplitudes (Fig. 1C a and b). An additional positive deflection with a long latency (22–35 ms) was usually recognized (Fig. 1A c and d, inverted filled triangles) at relatively high stimulus intensities that produced a clear PS of high amplitude. PS latencies as a function of stimulus intensity are shown in Fig. 1D. The PS latency decayed exponentially as the stimulus intensity increased. Because of the positive correlation between the stimulus intensity and PS amplitude, the PS latencies also decayed exponentially as a function of the PS amplitude (Fig. 1E a and b).

### Paired-pulse responses under the non-drug condition

**Figure 2:**
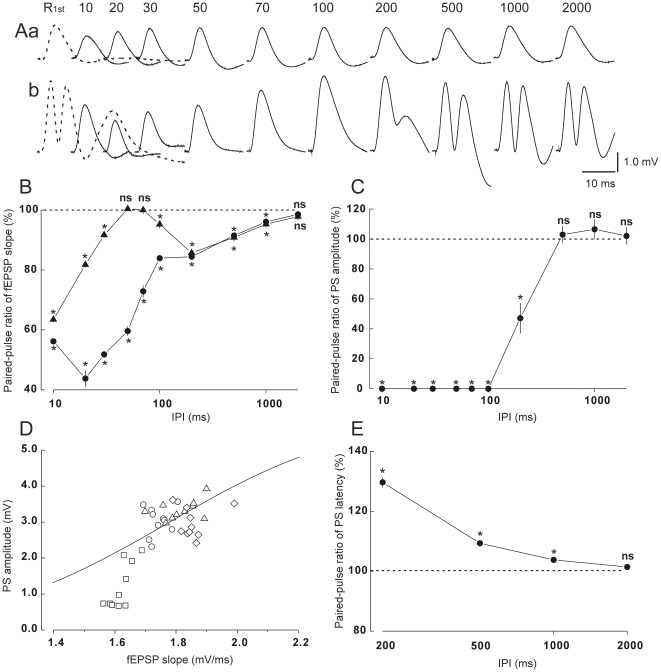
Paired-pulse responses under the non-drug condition. A: waveforms of response to the first stimulation pulse (R_1st_, broken line) and that to the second stimulation pulse (solid lines) at each inter-pulse interval (IPI) with low (a) or high (b) intensity stimulus. The numbers at top line, IPIs. Each waveform is an average of 10 LFPs. B: paired-pulse ratios for fEPSP slopes at low (triangles) or high stimulus intensity (circles) as a function of IPIs. C: paired-pulse ratios for PS amplitudes as a function of IPIs. D: relationships between fEPSP slopes and PS amplitudes in response to the second stimulation pulse at IPI of 200 (squares), 500 (circles), 1000 (triangles) and 2000 ms (diamonds). E: paired-pulse ratios for PS latencies as a function of IPIs. In B, C and E, vertical lines through each data point indicate standard error of means, and horizontal broken lines indicate paired-pulse ratio of 1 (100%). Other descriptions as for Fig. 1. Asterisks, *p*<0.05 in paired *t*-tests.

Figure 2A shows typical waveforms of the paired-pulse responses at different IPIs. Quantitative relationships between IPIs and the paired-pulse ratios of fEPSP slopes are shown in Fig. 2B. When the stimulus intensity was low (Fig. 2 Aa and triangles in Fig. 2B), a strong PPD was observed at the shortest IPI of 10 ms (*t* = 53.1, *df* = 9, *p*<0.05). This depression diminished as the IPI increased and was extinguished at IPIs of 50 and 70 ms (*t* = 0.44 and *t* = 0.04, respectively, *df*s = 9, ns). When the IPI was further increased, a weak but significant PPD again appeared, which gradually diminished at longer IPIs (*p*s<0.05 at IPIs of 100–1000 ms; ns at an IPI of 2000 ms). When the stimulus intensity was high (Fig. 2 Ab and circles in Fig. 2B), a PPD of a similar magnitude to that produced by the low intensity stimulation occurred at an IPI of 10 ms (*t* = 35.3, *df* = 9, *p*<0.05), and a further stronger depression appeared at an IPI of 20 ms (*t* = 22.3, *df* = 9, *p*<0.05). When the IPI was increased, the depression decreased through two phases, an early steep (IPIs of 30–100 ms; *p*s<0.05) and a late gradual (IPIs of 100–1000 ms; *p*s<0.05) phase.

Quantitative relationships between IPIs and the paired-pulse ratios of PS amplitudes are shown in Fig. 2C. The first pulses of the high intensity stimulation completely blocked PSs to the second pulse (2ndPS) at IPIs between 10 and 100 ms, and strongly depressed them at an IPI of 200 ms (Fig. 2 Ab and C; *t* = 4.81, *df* = 9, *p*<0.05). In contrast, the amplitudes of the 2ndPSs were almost the same as the PSs to the first stimulation pulse (1stPSs) at longer IPIs (*t*s = 0.25, 0.003 and 0.77 at IPIs of 500, 1000 and 2000 ms, respectively, *df*s = 9, ns). At IPIs between 200 and 2000 ms, the relationships between fEPSP slopes and 2ndPS amplitudes are shown in Fig. 2D, in which a part of the fitting curve shown in Fig. 1C is superimposed. All the data points at an IPI of 200 ms (squares) were located below the fitting line. At IPIs of 500 and 1000 ms (circles and upright triangles, respectively), more data points appeared above the fitting line, although these points were located adjacent to the line. These results indicate that the input (fEPSP)-output (2ndPS) ratio was decreased at an IPI of 200 ms, but tended to increase at IPIs of 500 and 1000 ms. Figure 2E shows the relationship between IPIs and latencies of 2ndPSs. The latency of 2ndPSs was considerably longer (up to 30%) than that of 1stPSs at an IPI of 200 ms (*t* = 18.6, *df* = 9, *p*<0.05). Although this prolongation of PS latency diminished as the IPI increased, the 2ndPS latencies at IPIs of 500 and 1000 ms were still significantly longer than those of 1stPSs (*t*s = 12.9 and 5.26, respectively, *df*s = 9, *p*s<0.05).

### Influence of diazepam on paired-pulse responses

The low dose (1.5 mg/kg) of diazepam moderately sedated the monkey, while the high dose (3.0 mg/kg) induced strong sedation. The sedative effect started to appear a few minutes after the administration of diazepam and remained at almost the same level for more than 1 hour.

**Figure 3:**
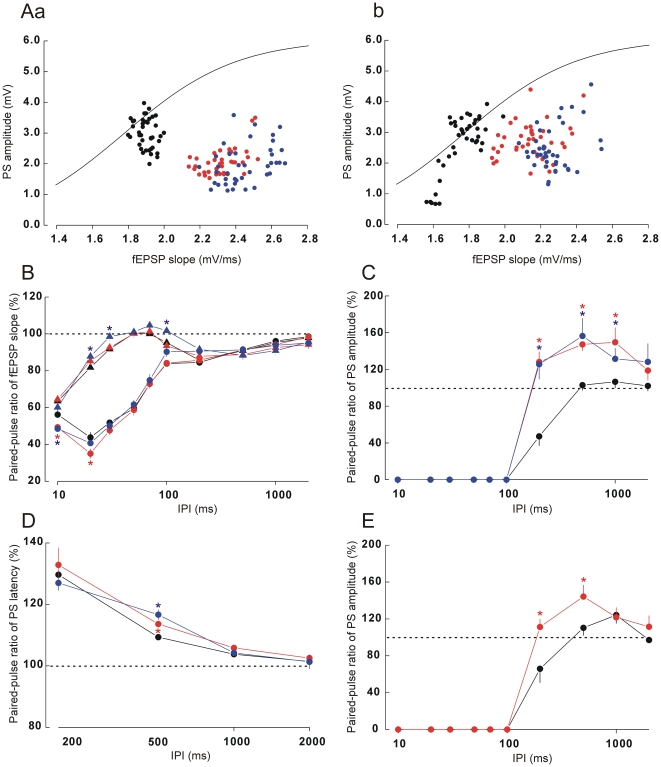
Influence of diazepam on paired-pulse responses. A: relationships between fEPSP slopes and PS amplitudes in response to the first (a) or second (b) stimulation pulses under control and treatment (low-dose and high-dose diazepam) conditions. Curve lines, a part of the sigmoid fitting line shown in Fig. 1C, which is calculated from the data on stimulus intensity-response relationship. B: paired-pulse ratios for fEPSP slopes at low (triangles) or high (circles) stimulus intensity as a function of IPIs. C, D and E: paired-pulse ratios for PS amplitudes (C), PS latencies (D) and PS amplitudes of balanced data subsets (E), respectively, as a function of IPIs. The subsets of data in E were extracted so that 1stPS amplitudes were comparable between the control and low-dose diazepam conditions. Black, red and blue symbols in each panel indicate data acquired under control, low-dose diazepam and high-dose diazepam conditions, respectively. Asterisks, *p*<0.05 in Dunnet pairwise comparisons after one-way ANOVA in B–D, and *p*<0.05 in one-way ANOVA in E. Other descriptions as for the Figs. 1 and 2.

For evoked LFPs to the first pulse with the low current intensity, both doses of diazepam slightly but significantly increased the fEPSP slopes in a dose-dependent manner (0.68±0.0019, 0.76±0.0023 and 0.80±0.004 mV/ms under control (non-drug), low-dose diazepam and high-dose diazepam, respectively; one-way ANOVA, F[2,27] = 466.3, *p*<0.05). When the high current intensity was used, the increases in fEPSP slopes under the treatment conditions were more obvious (1.90±0.0046, 2.33±0.0044 and 2.45±0.0056 mV/ms under the control, low-dose diazepam and high-dose diazepam, respectively; F[2,27] = 3461, *p*<0.05). In spite of these increases in fEPSP slopes, diazepam dose-dependently reduced 1stPS amplitudes (3.00±0.035, 2.26±0.080 and 1.98±0.037 mV under the control, low-dose diazepam and high-dose diazepam, respectively; F[2,27] = 91.7, *p*<0.05). Figure 3Aa shows the relationships between fEPSP slopes and 1stPS amplitudes under the three conditions. The data points of this relationship under the control condition (black circles) appeared on and around the fitting line calculated in the analysis of stimulus intensity response relationships (Fig. 1C). Because diazepam increased the fEPSP slopes while it decreased the PS amplitudes, the data points of the treatment conditions (red and blue circles) were located in the lower right part of the graph and a clear separation was recognized between the data population under the control and treatment conditions. In the re-recording under the control condition which was performed a week after the final administration of diazepam, the drug effects were no longer detected (data not shown).

Figure 3B shows the relationships between IPIs and the paired-pulse ratio of fEPSP slopes under the control and treatment conditions. With the low intensity stimulus (triangles in Fig. 3B), paired-pulse ratios under the treatment conditions showed a similar patter to that under the control condition, although high-dose diazepam produced no PPDs at IPIs of 30 and 100 ms (paired *t*-test, *p*s>0.05) at which weak PPDs were induced under the other conditions. With the high intensity stimulus (circles in Fig. 3B), paired-pulse ratios under the treatment conditions again showed a similar pattern to that under the control condition, although diazepam produced a slightly stronger PPD at IPIs of 10 and 20 ms.

Clear effects of diazepam on 2ndPSs were observed. Although 2ndPSs at IPIs between 10 and 100 ms were completely blocked by the first stimulation pulse (which was similar to the control condition), significant PPFs appeared at IPIs from 200–1000 ms (Fig. 3C, *p*s<0.05). Figure 3Ab shows the relationships between fEPSP slopes and 2ndPS amplitudes under the three conditions. When compared with the distribution of data on 1stPSs shown in Fig. 3Aa, the data points of the treatment conditions in Fig. 3Ab were located closer to the fitting line, reflecting the increased paired-pulse ratios under the treatment conditions. Figure 3D shows the relationships between IPIs and paired-pulse ratios of PS latencies under the three conditions. A similar trend of changes in 2ndPS latencies as a function of IPI appeared under the three conditions, although the values at an IPI of 500 ms under the treatment conditions were significantly higher than that under the control condition (F[2,27] = 9.39, *p*<0.05; post-hoc test, *p*s<0.05). This indicates that 2ndPSs had longer latencies in spite of the higher amplitude under the treatment conditions when compared with the control condition.

The fact that diazepam had a suppressive effect on PSs to the first stimulation pulses, so that the effect of recurrent inhibition may have been smaller when the response to the second pulse was elicited, might offer a simple explanation for the increase in paired-pulse ratios at longer IPIs. To address this issue, we extracted subsets for which the data of 1stPS amplitudes were comparable between the control and low-dose diazepam conditions (data on high-dose diazepam were not available because of its stronger suppressive effect on PS amplitudes). As shown in Fig. 3E, even in these data sets, the paired-pulse ratios of PSs were still increased at IPIs of 200 and 500 ms under the treatment condition compared with the control condition, although the difference was statistically marginal at the IPI of 500 ms (IPI of 200 ms: F[1,8] = 13.4, *p*<0.05; IPI of 500 ms: F[1,5] = 5.92, *p* = 0.059).

## Discussion

### Recording of paired-pulse responses in the primate hippocampus

Many paired-pulse experiments have been performed using the hippocampus of rodents. However, as far as we know, there have been no reports on paired-pulse responses in the monkey hippocampus *in vivo.* One main reason, among others, that this kind of experiment has not been performed using the monkey is that the hippocampus is a deep telencephalic structure of the primate brain and, therefore, precise positioning of electrodes is difficult without an appropriate guide. Also, the monkey's prehensile abilities make it difficult to perform LFP recording under a freely-behaving condition. We recently developed a method for recording evoked LFPs in the dentate gyrus of minimally restrained, freely-behaving monkeys [18], which led us to perform the present study.

There have been reports on paired-pulse responses in human epileptogenic hippocampus *in vivo* [21] and *in vitro* [22]. However, direct comparisons between the findings in these studies of human brain tissue and those studies using rodents do not necessarily make much sense because of the large methodological differences between them, including differences in the experimental (stimulation and recording) configurations. Also, it is necessary to take into consideration the possible pathological changes in the hippocampus accompanied with epilepsy. In contrast, the experimental configuration of the present study was considerably similar to those studies using rodents: in freely-behaving animals, stimulation was given to the medial part of the perforant path and paired-pulse responses were recorded in the hilar region of the dentate gyrus. This may make it possible to compare the results more directly between the two animal species.

### Responses to paired-pulse stimulation under the non-drug condition

One method to characterize the electrophysiological properties of presynaptic terminals is the paired-pulse test [23], [24]. At a low stimulus intensity that does not elicit granule cell discharges, the presynaptic effects of this test can be observed without contamination of multisynaptic effects. For the IPI dependency of paired-pulse responses to medial perforant path stimulation, there is a line of evidence for a substantial depression in a wide range of IPIs in rats [7], [9], [11]. Consistent with these studies, the paired-pulse test of the present study revealed that PPD predominantly appeared in the dentate gyrus of monkeys when the medial part of the perforant path was stimulated. Therefore, as was previously indicated in rats [7], the medial part of the perforant path could release a relatively large fraction of its available transmitter per impulse in monkeys as well.

Paired-pulse tests with high intensity stimuli eliciting clear PSs have been used to evaluate the recurrent (and feed forward) inhibition in the dentate gyrus of rodents [12], [13], [16]. When local inhibitory interneurons in the dentate gyrus such as pyramidal basket cells [25] receive excitatory inputs from the granule cells so that they elicit impulses, these cells, in turn, give rise to a recurrent inhibition onto the granule cells. The response induced by the second pulse to the perforant path is thus depressed by this recurrent inhibition in a manner dependent on IPIs. Similar types of inhibitory interneurons to those in the rodent dentate gyrus are reportedly located in the monkey dentate gyrus [26], [27]. Therefore, this type of recurrent inhibition may also be involved in the depression of the fEPSP slope and blockage of 2ndPSs at the high stimulus intensity observed in the present study. The changes in the PPD of fEPSP slopes as a function of IPIs in the presence of 1stPSs were mostly comparable to those reported in rats [9], [11]. In contrast, changes in the paired-pulse effects on PS amplitudes as a function of IPIs seemed to be delayed in monkeys more than in rats. That is, IPIs with a complete blockage of 2ndPS appeared between 10–100 ms, and 2ndPS amplitudes depressed by *ca.* 50% at an IPI of 200 ms in the present study, while in rats, IPIs with complete blockage of 2ndPS usually appear at short IPIs (10–20 ms) and depression of 2ndPS remains up to *ca.* 50 ms [9], [11], [12].

### Effect of diazepam on paired-pulse responses

The paired-pulse test has frequently been used in the dentate gyrus of rats to characterize the effect of diazepam on GABAergic inhibition [12], [13], [17]. In the present study, the two doses of diazepam which produced a clear sedative action, slightly but significantly increased fEPSP slopes in response to the first pulse at the low current intensity in a dose-dependent manner. This increase in fEPSP slopes under the treatment conditions appeared more obviously when the high current intensity was used. In spite of the clear increase in fEPSP slopes, diazepam dose-dependently decreased the 1stPS amplitudes. These results indicate that diazepam reduces the input (fEPSPs)/output (PSs) ratio of granule cells. Previous studies testing the effect of diazepam on paired-pulse responses in the dentate gyrus of rodents have reported no significant changes in fEPSP slopes and PS amplitude in response to the first stimulation pulse [12], [13], [28] (but inhibition of PS amplitude [17]), which were inconsistent with the present findings.

Although no apparent effects of diazepam were observed on the complete blockage of 2ndPSs at IPIs between 10 and 100 ms, the PPD at the IPI of 200 ms observed under the control condition was reversed to PPF, and the PPFs at IPIs of 500 and 1000 ms were enhanced. In rodents, administration of diazepam is known to enhance the recurrent inhibition in the dentate gyrus so that PPD is enhanced and PPF is reversed to PPD [13], which are opposite to the present findings. Because diazepam had a suppressive effect on the amplitude of 1stPSs in the present study, the recurrent inhibition *per se* could have been reduced to some degree, which would have increased the paired-pulse ratios at longer IPIs. To address this issue, we extracted subsets of data on which the 1stPS amplitudes were comparable between the control and treatment conditions. Even in these data sets, the paired-pulse ratios of PSs were still increased at IPIs of 200 and 500 ms under the treatment condition compared with the control condition, although the difference became smaller. These results indicate that the suppressive effect of diazepam on 1stPSs only partially explains the increased paired-pulse ratios for PS amplitude, and therefore, additional mechanisms may also have contributed to this increase.

### Conclusions

This is the first report on characterization of paired-pulse responses in the dentate gyrus of monkeys *in vivo*. The results indicate that paired-pulse responses in the monkey are similar to those of rodents in many aspects, although PSs in the monkey are inhibited for a longer period than those in rodents once they occur and have a different sensitivity to diazepam. Although the core of the present method is not novel, it is reliable and stable and can widely be applicable to various experiments of the primate hippocampus *in vivo*.
